# 基于电泳滴定和电容耦合非接触电导检测测定人源血清总蛋白

**DOI:** 10.3724/SP.J.1123.2023.04015

**Published:** 2023-08-08

**Authors:** Ruihua ZHANG, Zehua GUO, Qiang ZHANG, Genhan ZHA, Chengxi CAO, Liuyin FAN, Weiwen LIU

**Affiliations:** 1.上海交通大学电子信息与电气工程学院,上海 200240; 1. School of Electronic Information & Electrical Engineering, Shanghai Jiao Tong University, Shanghai 200240, China; 2.上海交通大学学生创新中心,上海 200240; 2. Student Innovation Center, Shanghai Jiao Tong University, Shanghai 200240, China

**Keywords:** 电泳滴定, 电容耦合非接触电导检测, 血清总蛋白, electrophoresis titration, capacitively coupled contactless conductivity detection, serum total protein

## Abstract

血清总蛋白含量检测与人体健康监测和疾病诊断密切相关,本文基于电泳滴定(ET)技术结合电容耦合非接触电导检测(C^4^D)技术实时捕获ET过程中通道内物质的电导率变化,在不依赖指示剂和光学检测设备的情况下,定量检测人源血清总蛋白含量,ET-C^4^D检测总耗时约300 s。本文以人源血清白蛋白(HSA)标准品作为模式蛋白,与聚丙烯酰胺凝胶(PAG)母液、核黄素等混合后,在紫外灯光下照射10 min聚合形成凝胶,进行ET实验,耦合在ET管道外壁的非接触式电导检测电极捕捉通道内物质在电泳过程中的电导率信号,经检测模块和数据采集卡处理后送入计算机,由开发的分析测试软件根据电导率信号进行定量分析,结果表明:线性范围为0.25~3.00 g/L,线性拟合度(*R*^2^)在0.98以上,检出限(LOD)为0.01 g/L,相对标准偏差为1.90%,0.50 g/L的HSA标准品的测试值与实际值的相对误差低于7.20%,表明该检测系统具备较好的检测稳定性和灵敏度。最后,针对人源实际血样中的血清总蛋白含量进行检测,建立了相应的总蛋白标准曲线,然后选取4位志愿者的血清样本进行ET-C^4^D检测,并将ET-C^4^D检测结果与双缩脲法的检测结果进行了比对,两种方法检测结果的相对误差在4.43%以内,进一步证明了该检测系统的准确性与可用性,以及该检测系统在临床即时检测(POCT)领域的潜在应用价值和生化分析价值。

## Abstract (en)

Serum total protein refers to the sum of all proteins in the serum, and its content determination is relevant to human health monitoring and disease diagnosis. However, existing detection techniques present a number of limitations; for example, the Kjeldahl method suffers from the negative effects of interfering substances such as non-protein nitrogen (NPN). Although the electrophoresis titration (ET) method has solved interference problems to some extent, the current ET technique relies on optical detection methods, which increases the tediousness of the operation.

This study addresses the challenge of accurate serum total protein detection by combining the traditional ET technique with capacitively coupled contactless conductivity detection (C^4^D). The research contributions of this work are multifold. First, it presents the first development of an ET-C^4^D detection system, which consists of six components: an ET power module, an ET chip, a C^4^D sensing module, a detection module, a data acquisition card, and software. The developed system can capture the conductivity of substances in the channel using the software developed by our laboratory during ET. The detection system can be used to quantify the total protein content in human serum without the addition of specific labeling reagents or using optical detection equipment, and its running time is approximately 300 s. Second, this research proposes the corresponding principle of the system. Under an electric field, ion migration results in different pH levels before and after the boundary, leading to a protein surface charge difference. The maintenance of the electrical neutrality of the substances in the detection channel is related to the protein surface charge; therefore, the ion concentration distribution of the substances in the detection channel changes as the protein surface charge varies. A plot of conductivity as a function of running time showed an “inverted clock shape”, first falling and then rising. Owing to the addition of different types and concentrations of proteins, the microenvironment of the entire system changes, resulting in different changes in conductivity. Third, the performance of the detection system was tested using human serum albumin (HSA) standard protein, which was mixed with polyacrylamide gel (PAG) mother liquor, riboflavin, etc., and irradiated under ultraviolet light for 10 min to form a gel. The ET experiments were then carried out. The shape of the conductivity curve was consistent with the proposed principle, and the higher the HSA concentration, the lower the conductivity curve trough, followed by a lagged time of the trough. Quantitative analysis of the conductivity signals showed that the linear range was 0.25-3.00 g/L, with a linearity of up to 0.98. The limit of detection (LOD) was 0.01 g/L, the relative standard deviation (RSD) was 1.90%, and the relative error of the test values was <7.20%, indicating the good detection stability and sensitivity of the system.

Clinical samples collected from healthy volunteers were used as target blood samples for serum total protein content measurement using our detection system. Blood samples from a volunteer were used to obtain a standard curve, and the serum samples of other four volunteers were selected for ET-C^4^D and biuret detection. The results showed that the relative errors between the two methods were within 4.43%, indicating the accuracy and reliability of the detection system. The advantages of the ET-C^4^D detection system proposed in this paper are as follows: (i) ET-C^4^D realizes the rapid detection of total serum protein content based on the ET technique; (ii) compared with the traditional protein ET technique, the ET-C^4^D method does not rely on specific labeling components or optical detection equipment, thereby reducing the complexity of the operation; and (iii) the output signal of ET-C^4^D can be used for quantitative analysis with excellent analytical performance and high accuracy. These merits highlight the potential of the developed system for clinical application and biochemical analysis.

血清总蛋白是血清中所有蛋白质的总称,在维持血液酸碱度、调节人体水分平衡等环节扮演着重要的角色^[1,2]^。血清总蛋白含量偏离正常区间范围易导致多发性骨髓瘤、甲状腺功能亢进、肝硬化等疾病^[3]^,其含量的准确测定与人体的健康监测及此类疾病诊断密切相关^[3,4]^。目前已经发展出许多技术用于总蛋白含量的测定,包括凯式定氮法^[4,5]^、双缩脲法^[6]^、杜马斯燃烧法^[5]^和光谱分析法^[7,8]^等。

凯式定氮法是测定总蛋白含量的经典方法^[5]^,其虽然具有良好的普适性,但是操作流程复杂,需要在催化剂作用下使用硫酸消解样品,此类腐蚀性试剂的使用常存在潜在安全隐患,且凯式定氮法无法解决非蛋白氮(non-protein nitrogen, NPN)的干扰问题,这对总蛋白含量的定量造成了一定影响^[9]^;双缩脲法是血清中总蛋白含量测定的常规方法^[3,10]^,其通过加热样品形成双缩脲,在碱性条件下与二价铜离子作用产生紫红色络合物,最后通过测量吸光度来测定血清总蛋白含量,然而,定量分析往往依赖专业光学仪器。

基于移动反应界面(MRB)理论的蛋白质电泳滴定(electrophoresis titration, ET)技术为上述问题提供了新的思路^[11⇓⇓⇓-15]^,其将蛋白质的浓度信号转变为MRB的位移信号,操作过程避免了腐蚀性试剂的使用,以及NPN干扰物质的负面影响,检测时间也快于传统方法。然而,最初的ET技术常需要依赖较大型的仪器^[16]^, Wang等^[17]^基于先前的工作提出了双内标-电泳滴定检测模型(double inner standard plot (DISP) model of electrophoresis titration (ET), DISP-ET),可以实现乳品总蛋白含量的快速现场检测,但检测过程需要添加指示剂,浓度信号传感依赖于有色界面,结果分析需要光学设备辅助,增加了操作的繁琐程度。

电容耦合非接触电导检测(capacitively coupled contactless conductivity detection, C^4^D)自从1998年被Zemann等^[18]^提出后,被广泛应用于解决复杂生物样本的分析问题^[19,20]^,其成本低,样品消耗少,能够以非接触式方法在线检测生化反应过程^[21]^,有望为蛋白质ET检测分析提供一条新的途径。基于蛋白质ET技术与C^4^D技术,本文搭建了一种ET-C^4^D检测系统并提出了相应的定量计算方法,对人源血清白蛋白(human serum albumin, HSA)标准品进行了检测分析,并对从医院获得的真实血清样本的总蛋白含量进行了检测,验证了检测系统的有效性和准确性,也证明本文提出的检测系统在临床分析中具有潜在应用价值。

## 1 实验部分

### 1.1 ET-C^4^D检测系统

整个ET-C^4^D检测系统的示意图如图1所示,它由6个部分组成,从左到右分别是:ET电源模块、ET芯片、C^4^D传感单元、检测电路模块、数据采集卡和分析测试软件。ET电源模块可以提供0~250 V的电压来运行电泳,在这套检测系统中,采用的是150 V的电压。ET芯片用于ET和电导检测,它由聚碳酸酯板制成,包括一个阴极井和一个阳极井。C^4^D传感单元通过一条柔性扁平电缆与检测电路模块相连,用于采集通道内物质的电导率信号,它包括一个短的玻璃毛细管、一个激励电极和一个检测电极,其中玻璃毛细管总长度为5 cm,外径为1.2 mm,内径为0.6 mm;电极是铜管,长度为1 cm,外径为1.8 mm,内径为1.3 mm,耦合在玻璃毛细管道外壁,两个电极之间的距离为1 cm。检测电路模块由激励信号源、电流/电压(*I/V*)转换电路和峰值整流电路组成,激励信号源产生频率为110 kHz、峰-峰值为20 V的交流激励信号,检测电极拾取的电流信号经*I/V*转换和放大输出电压信号,后经峰值整流电路转变为直流信号接入数据采集卡进行数字化。分析测试软件为实验室自主开发的多通道非接触电导测量分析软件^[22]^,将计算机端采集的输出电压信号转换为电导率信号并进行定量分析。

**图1:**
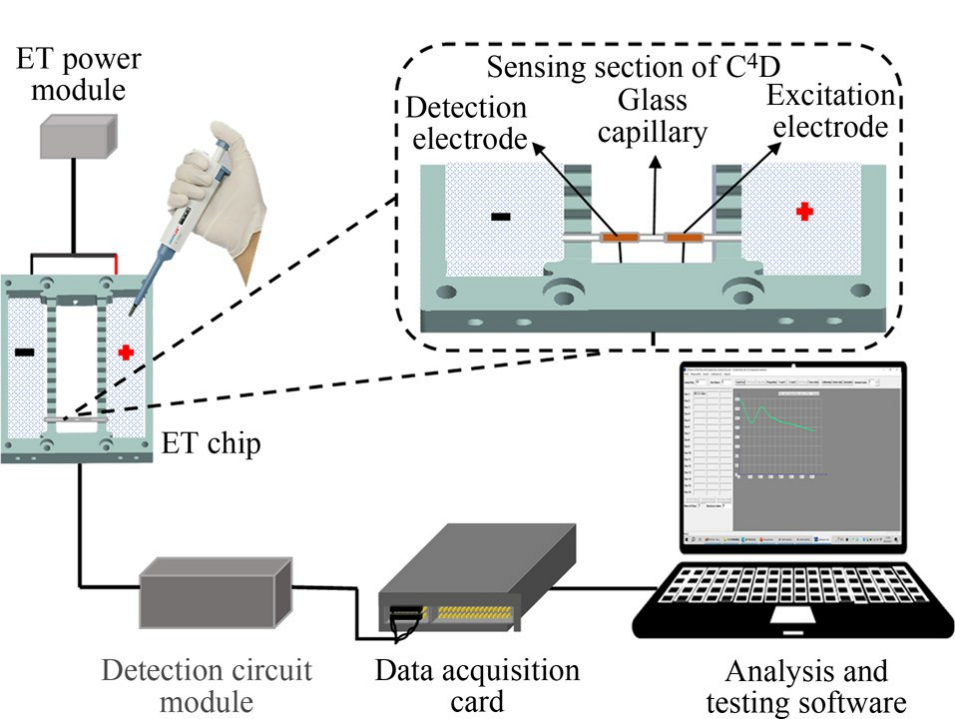
ET-C^4^D检测系统的示意图

### 1.2 仪器和材料

实验采用高速离心机(Beckmann,美国)进行血清样本的提取,采用Pall超滤浓缩离心管(Nanosep,美国)进行血清样本的除盐处理,采用AU5811全自动生化分析仪(Beckmann,美国)进行血清总蛋白含量的双缩脲法检测,采用ML204电子天平(梅特勒托利多,瑞士)进行样品称量,采用16~20 W的紫外灯(365 nm)进行聚丙烯酰胺凝胶(polyacrylamide gel, PAG)聚合,采用MP417数据采集卡(北京双诺测控技术有限公司)采集ET过程中的电导率变化信号,采用惠普电脑(美国)进行数据记录和处理。

双丙烯酰胺(bis-acrylamide)购自国药化学试剂有限公司;氯化钾、氢氧化钠和盐酸购自凌峰化学试剂有限公司;氢氧化钾和丙烯酰胺(acrylamide)购自上海麦克林生化科技有限公司;四甲基乙二胺(tetramethyl ethylenediamine, TEMED)购自生工生物工程有限公司;维生素B2(核黄素)购自Sigma-Aldrich公司;HSA标准品购自上海碧云天生物技术有限公司。所有化学试剂均为分析纯,无需进一步纯化处理,所有溶液均使用SZ-93自动蒸馏器(中国亚荣)制得的去离子水制备。

实际血样取自上海市第六人民医院(临港院区),本研究已通过上海交通大学附属胸科医院临床研究伦理委员会审批,批准号:KS2011。

### 1.3 溶液制备

阴极液:200 mmol/L NaOH;阳极液:20 mmol/L KCl; ET通道含有12 μL固定液;模式蛋白母液:制备100 g/L的HSA溶液,储存在4 ℃的冰箱备用;PAG母液:28.8 g的丙烯酰胺和1.2 g双丙烯酰胺溶于水,定容至100 mL。

固定液制备:ET过程借助凝胶将大分子蛋白质固定在通道中,本文采用光聚合法聚合凝胶来固化蛋白质。第一步是制备5 mL的凝胶母液:包括3.125 mL的PAG母液、40 μL酚酞(1%,质量分数)、62.5 μL KCl母液(200 mmol/L)、25 μL饱和核黄素溶液(0.12 mg/mL)和0.2 μL TEMED。第二步是制备含有不同待测蛋白质的固定液:取0~20 μL的HSA母液(100 g/L水溶液)与320 μL的凝胶母液混匀,得到含有HSA浓度梯度(0.01、0.05、0.25、0.75、1.5、3、5 g/L)的固定液。

血液样本前处理:使用EDTA抗凝管采集静脉血5 mL,将血液在离心机中以4000 r/min的转速离心5 min后,吸取上层清液,然后将上清液加入超滤浓缩离心管以5000 r/min的转速离心20 min,去除滤液,向超滤管中加入与滤液等体积的2 mmol/L KCl溶液,保存在-20 ℃冰箱中备用。

### 1.4 双缩脲法

参考文献[23],对采集得到的静脉血在离心机中以4000 r/min的转速离心5 min,提取上层清液,用双缩脲法测得5位志愿者样本的血清总蛋白质量浓度分别为71.0、73.0、75.0、77.0、78.0 g/L。

### 1.5 ET-C^4^D检测原理和定量计算方法

取12 μL固定液填充到ET管道中,紫外光下均匀照射10 min形成凝胶。凝胶固化后,施加ET电压,开始ET电导信号监测。ET-C^4^D的原理和输出信号示意如图2所示:在图2a中,通电前,蛋白质被均匀地固定在电泳通道中,通电后,在电场作用下阴极池中的OH^-^向阳极端迁移,替换原背景液中的Cl^-^,但迁移过程中蛋白质消耗掉一部分OH^-^,同时K^+^向阴极端迁移,使得ET界面前逐渐形成一段低离子浓度区,当低离子浓度区进入电导检测区,电导率信号持续下降;但随着ET逐步进行,由于OH^-^完全置换了原有的Cl^-^, ET结束区域的pH急剧上升导致蛋白质表面电荷增大,同时该区域的K^+^浓度迅速上升以维持电中性,原有的离子低浓度区被离子高浓度区逐步取代,电导率继而回升,整个变化的电导率趋势如图2b中所示。不同含量的蛋白质具有不同数量的滴定位点,导致下降沿呈现不同的幅度,根据ET原理^[11]^可知,蛋白质浓度越高,界面迁移速率越慢,电导率下降至波谷的时间也会随之滞后,因而后续可以借助下降曲线数据来定量待测蛋白质的浓度。

**图2:**
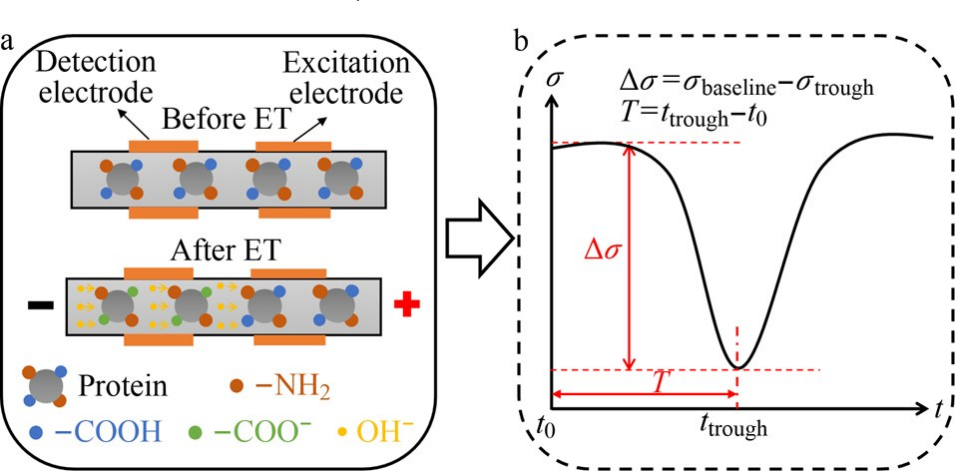
ET-C^4^D的(a)原理和(b)输出信号示意图

图2b呈现了ET-C^4^D的定量计算方法,其中Δ*σ*代表电导率基线值和波谷的差值,*T*代表电导率波谷所对应的时间,在之后的实验应用中可以利用上述物理量,通过绘制标准曲线,来量化人血清总蛋白含量。

## 2 结果与讨论

### 2.1 HSA的检测

HSA是血清总蛋白中占比最高的一种成分,也是多种疾病(癌症、肝脏疾病^[24]^等)的有效生物标志物,本文采用HSA对ET-C^4^D的性能进行初步验证,图3呈现了PAG体系下HSA的相应检测结果,图3a中不同浓度的HSA呈现出形状相似但幅度不同的电导率变化趋势:每个信号均呈现“倒钟形”,变化趋势为先降后升再趋于平缓。且HSA浓度越高,下降的幅度越大,波谷出现的位置也随之滞后;当HSA浓度继续升高时,电导率持续下降,固定的检测窗口无法检测到电导率回升信号。

**图3:**
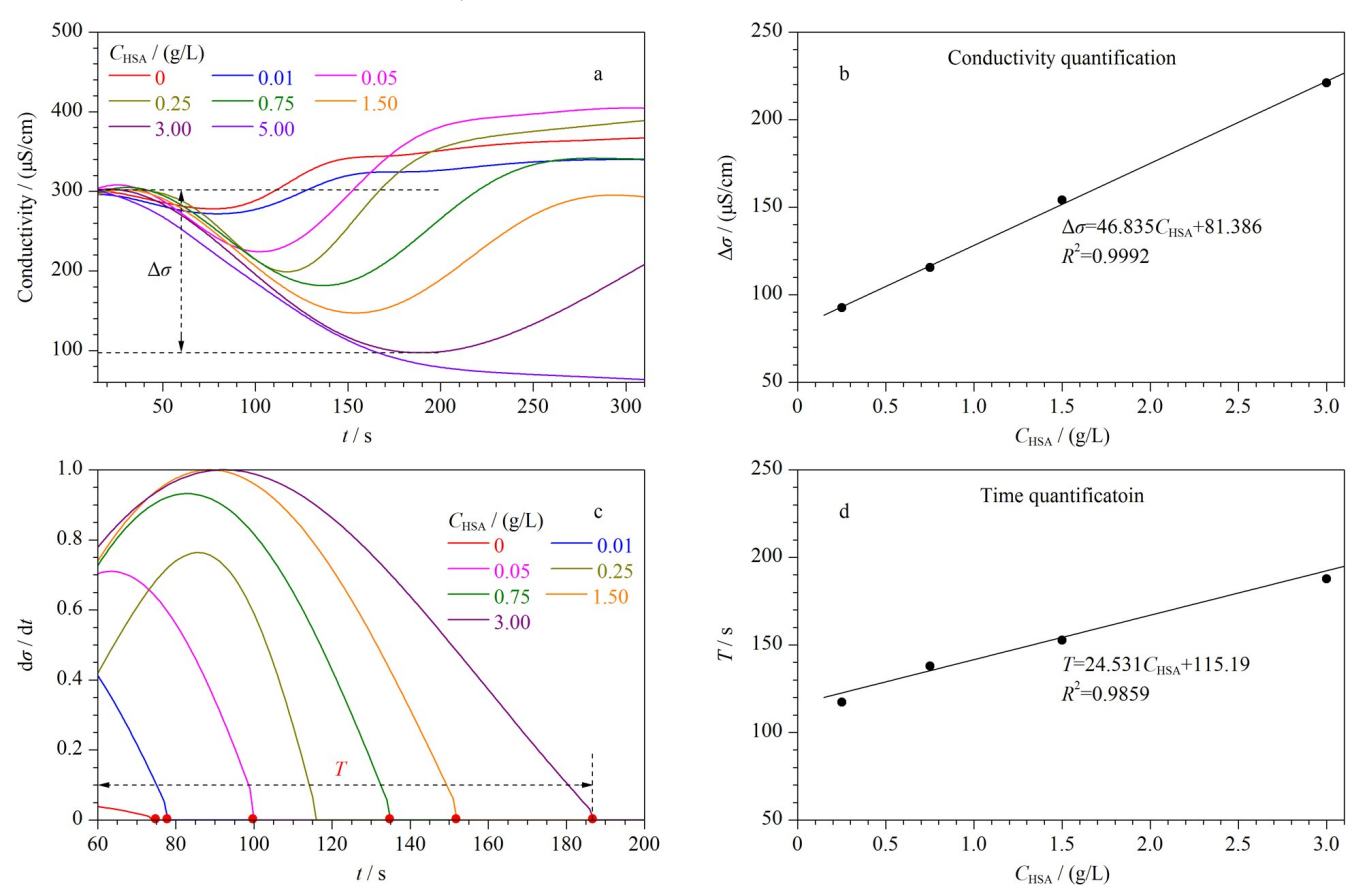
采用HSA对检测系统进行性能测试的结果

图3c是针对图3a曲线求导得到的,以此来定位波谷出现的时刻。图3b和3d是分别针对*Δ*σ和*T*(见图1)对HSA浓度进行定量得到的线性曲线,可以看到在一定范围内两种定量方式均呈现良好的线性关系,从而区别于传统的ET可视化定量法。

表1分析了图3b和3d中电导率定量法和时间定量法的性能,可以看出,它们的线性范围均为0.25~3 g/L,线性相关系数(*R*^2^)分别为0.9992和0.9859以及RSD(*n*=3)分别为1.90%和1.30%;将HSA浓度逐步稀释可以得到此检测系统测试的检出限(LOD)为0.01 g/L。得到两定量方法的标准曲线后,采用0.5 g/L的HSA对其进行了测试,检测得到的结果与真实值进行比对后相对误差如表1所示,验证了ET-C^4^D检测系统对于HSA较好的分析性能。

**表1:** ET-C^4^D电导率定量法和时间定量法测量HSA的线性范围、稳定性与LOD

Method	Linear range/ (g/L)	RSD/% (*n*=3)	LOD/ (g/L)	Relative error^*^/%
Conductivity	0.25-3.00	1.90	0.01	5.10
quantification				
Time quantification	0.25-3.00	1.30	0.01	7.20

* HSA mass concentration: 0.5 g/L.

### 2.2 实际血样中血清总蛋白含量的定量测定

为了验证ET-C^4^D检测系统对实际血清样本总蛋白含量检测的有效性,首先选取志愿者血样(用双缩脲法测得的血清总蛋白质量浓度为78.0 g/L),梯度稀释后得到质量浓度为0.0780、0.195、0.390、0.780、1.17、1.56 g/L的样本,从而获得血清总蛋白的ET-C^4^D检测结果对应浓度的标准曲线(见图4)。图4a和4c中血清总蛋白与图3中HSA模式蛋白的ET-C^4^D测试结果呈现类似的形状与变化规律,表明检测系统对血清总蛋白具有检测能力。血清总蛋白与HSA模式蛋白并不完全相同,其为包含HSA在内的多种蛋白质的混合物,而不同种类的蛋白质在电泳过程中对离子迁移的影响程度不同,体现在图4b和4d给出的血清总蛋白拟合线性方程与HSA拟合线性方程的线性回归系数大小存在明显差异,这也是在此重新建立血清总蛋白标准曲线的原因。此外,本实验中实际血清样本在前期预先进行了除盐处理,因此电泳时血清样本中背景离子的影响忽略不计。

**图4:**
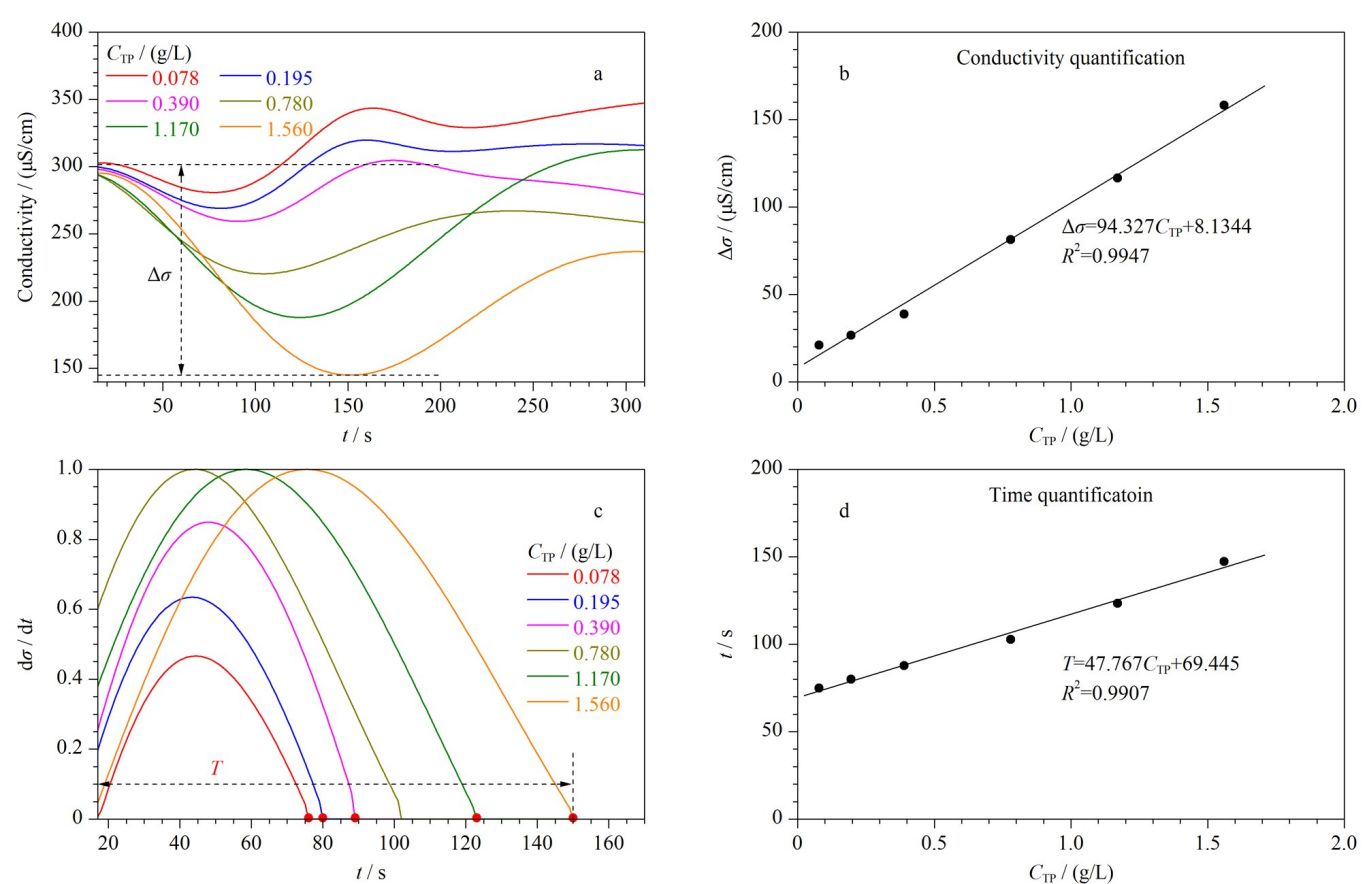
血清总蛋白的ET-C^4^D结果和标准曲线图

然后,选取其余4位志愿者的血清样本各稀释200倍进行ET-C^4^D检测并用所建立的标准曲线计算出浓度值,最后将其与用双缩脲法检测的结果进行比对。由于图4b中的电导率定量法计算过程更为简便,因此在实际测量计算中采用图4b的标准曲线进行计算。测试结果如表2所示,可以得知ET-C^4^D对血清总蛋白检测的相对标准偏差为2.09%~3.23%,与双缩脲试剂标准法的测量结果相对误差为1.52%~4.43%。该结果证明ET-C^4^D检测系统具有较高的准确性和稳定性,并且能够成功地应用到实际血清样本的定量检测。

**表2:** 临床血清样本检测结果

Serum sample	Biuret reagent standard method/ (g/L)	ET-C^4^D method/ (g/L)	RSD/% (*n*=3)	Relative error/%
1	71	67.86	2.27	4.43
2	73	71.89	2.15	1.52
3	75	73.23	3.23	2.36
4	77	73.90	2.09	4.02

## 3 结论

本文提出了一种基于ET技术和非接触电导检测技术的ET-C^4^D检测系统,用于HSA及人血清总蛋白的检测。结果表明,该检测方法呈现出较好的分析性能,耗时短,可以在300 s以内实现蛋白质的快速检测,且具备较高的灵敏度和稳定性;通过对真实的人源血清样本进行检测并计算其中总蛋白的含量,证明了该方法的临床应用价值。与传统方法相比,该方法操作简便,无需添加特定标记试剂,也不依赖专业光学检测设备,检测结果可以用于定量分析,分析性能优异,准确度高,在生物分析领域具有很高的应用潜力。
